# Spontaneous bone regeneration in segmental mandibular defects: A systematic review

**DOI:** 10.4317/medoral.26712

**Published:** 2024-08-01

**Authors:** Tai Wei, Ying-Ying Fan, Shu Li, Ye-Jun Cai, Xin Peng, Peng Ye

**Affiliations:** 1 PhD, Peking University School and Hospital of Stomatology First Clinical Division; 2 DDS, Department of Stomatology, Beijing Hospital, National Center of Gerontology, Institute of Geriatric Medicine, Chinese Academy of Medical Sciences; 3 MD, Department of Stomatology, Beijing Hospital, National Center of Gerontology, Institute of Geriatric Medicine, Chinese Academy of Medical Sciences.; 4 DDS, Department of oral and maxillofacial surgery, Peking University School and Hospital of Stomatology

## Abstract

**Background:**

Rehabilitation for segmental mandibular defect is vital for mastication function and facial aesthetics. Interestingly spontaneous bone regeneration after segmental mandibulectomy sporadically occurs to avoid further bony rehabilitation. This study aimed to assess the potential of spontaneous bone regeneration in the treatment of mandibular defects.

**Material and Methods:**

An electronic search was conducted using the PubMed, EMBASE, Wiley Online Library, and Cochrane Library databases to identify eligible studies. Critical appraisal of the included articles was done using the Joanna Briggs Institute critical appraisal checklist.

**Results:**

A total of 35 studies, including 60 patients, that investigated spontaneous bone regeneration after segmental mandibulectomy were included. Among these patients, 39 (65%) were male and 21 (35%) were female, with a mean age of 20.81 ± 16.38 years. Periosteum was completely and partially preserved during mandibulectomy in 25 and 13 patients, respectively. Continuous bone regeneration between mandibular stumps was observed in 53 (88.3%) patients during follow-up. Although the mandibular stump was not stabilized in 13 (21.67%) patients, continuous bony regeneration still occurred, with a mean recovery period of 30.29 months. This was significantly greater than the overall average recovery time of 19.87 months.

**Conclusions:**

Spontaneous bone regeneration could occur in segmental mandibular defects, particularly in young patients with intact periosteum and rigid mandibular stump fixation.

** Key words:**Bone regeneration, treatment, mandibular defect, systematic review.

## Introduction

Critical sized defects are bony defects that do not heal spontaneously, requiring surgical intervention (1), and are derived from a variety of pathological causes, including oral cancer, trauma, and infections. Segmental mandibular defects are commonly critical sized, and their reconstruction poses a challenge for oral and maxillofacial surgeons. Mandibular rehabilitation is vital for mastication, speech, and facial aesthetics, particularly in young patients. Free bone grafts harvested from the iliac crest or rib are commonly used to reconstruct mandibular defects. However, an uneventful recovery cannot be assured in cases requiring adjuvant radiation and those with defects greater than 5 cm. Pedicled bone grafts, including the vascularized fibula free flap, have superior osteogenic capacity in large mandibular defects but have a lower growth potential compared to the contralateral mandibular stump (2). This may result in facial deformities in the long term, restricting the use of pedicled bone grafts in mandibular reconstruction. Interestingly, spontaneous bone regeneration has been observed in a few pediatric and adult patients who underwent mandibulectomy without simultaneous rehabilitation. Secondary reconstructive surgery could even be avoided in a few cases (3-4).

The present review analyzed the demographic and clinicopathological data of patients undergoing spontaneous bone regeneration after segmental mandibulectomy and evaluated the potential of spontaneous bone regeneration as an alternative treatment for segmental mandibular defects.

## Material and Methods

- Registration and protocol

A comprehensive search for reviews on the topic was conducted in the International Prospective Register of Systematic Reviews (PROSPERO) database, but no relevant studies were identified. The protocol for our systematic review was registered with PROSPERO (No. CRD42023439100).

The Preferred Reporting Items for Systematic Reviews and Meta-Analyses (PRISMA) statement was used as a guide for reporting the results of this systematic review.

- Eligibility criteria

Inclusion criteria: Based on the PICOS principle, the inclusion criteria for this systematic review were: (P) patients with segmental mandibular defects; (I) mandibulectomy without simultaneous bony reconstruction; (C) not applicable; (O) spontaneous bone regeneration; and (S) case reports and case series.

Exclusion criteria: The exclusion criteria were reviews, letters to the editor, unpublished data, and articles without a clear description of the outcome for segmental mandibular defects.

- Information sources

Comprehensive electronic searches without any date restrictions were conducted up to August 2023 using the Cochrane Library, PubMed, EMBASE, and Wiley Online databases.

- Search strategy

Search terms included the following Medical Subject Heading (MeSH) terms and free text words: “mandible” and “bone defect” in combination with “bone regeneration” and “spontaneous.” The references of the included studies were manually searched for potential studies that could be included.

 -Study selection

Titles and abstracts of the studies were independently reviewed by two authors (T.W, Y.Y.F). Full-text evaluation was subsequently performed if the title and abstract met the eligibility criteria. In case of any discrepancies, a fourth reviewer was consulted to achieve a consensus.

- Data extraction

For each included study, the following details were collected: publication details (first author and year of publication), patient details (sex and age), pathological diagnosis, type of mandibulectomy, operative approach, periosteal status, mandibular stump stabilization, bone regeneration, and the follow-up period. Incomplete data were indicated as “not mentioned” (N.M.).

- Study risk of bias assessment

The Joanna Briggs Institute (JBI) critical appraisal checklist for case reports was used independently by two authors (Y.Y.F and T.W) for critical appraisal of the selected articles (5). The following eight domains were evaluated for each article: clear description of patient demographic characteristics; presentation of the patient’s history in a chronological timeline; clear depiction of the clinical condition upon presentation; detailed description of diagnostic tests and assessment methods, including their results; clear description of intervention(s) and treatment procedures; clear depiction of the post-intervention clinical condition; identification and description of adverse and unanticipated events; and presence of takeaway lessons in the report.

- Data synthesis methods

Detailed quantitative synthesis of the results of the included studies was performed and presented in this systematic review.

## Results

- Study selection

**Figure 1:**
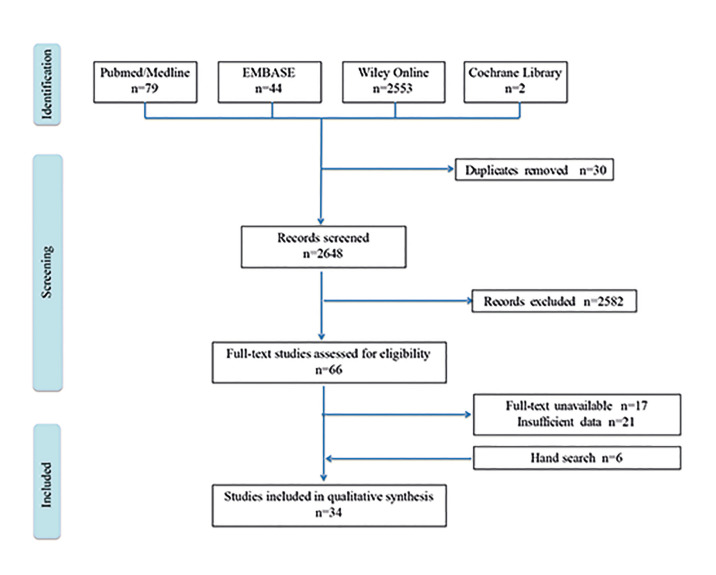
Flow diagram of the study selection process.

The initial comprehensive search yielded 2,678 articles. After removing 30 duplicates and implementing the inclusion and exclusion criteria, 66 articles were screened for full text. Unavailability of full text or insufficient data resulted in the exclusion of further 26 studies. A manual search of the references of the selected articles yielded six additional articles. Thus, a total of 34 articles were included in the final qualitative synthesis (3-4,6-37). The study selection process is illustrated in Fig. 1.

- Demographic and clinicopathological data

The included articles, published between 1946 and 2022, comprised 27 case reports and seven case series. Sixty patients were reported in a total of 34 eligible studies, including 39 (65%) males and 21 (35%) females, with a male-to-female ratio of 1.86:1. The mean and median ages were 20.81 ± 16.38 years and 15 years, respectively, ranging from 8 months to 73 years.

The etiology of the mandibular defect was reported for all included cases. The defect was caused by a benign tumor in 34 (56.7%) cases, with ameloblastoma (33.3%) being the most frequent pathological diagnosis, followed by cemento-ossifying fibroma, giant cell granuloma, odontogenic myxoma, and osteofibroma. The defect resulted from a malignancy in 8 (13.3%) cases, with Ewing’s sarcoma (n = 3) being the most common pathological type, followed by myxofibroma, myxofibrosarcoma, hemangioendothelioma, and osteosarcoma. The defect was caused by trauma, medication-related osteonecrosis of the jaw (MRONJ), and osteomyelitis in 3, 2, and 2 cases, respectively.

The operative approach was described in 25 patients. Extraoral approach, intraoral approach, and their combination was used in 12 (48%), 8 (32%), and 5 (20%) cases, respectively. Based on the HCL classification of mandibular defects, 39 (65%), 12 (20%), and 15 (25%) cases involved L type, H type, and C type defects, respectively, while one case could not be categorized.

Periosteal status after the mandibulectomy was reported for 50 of the 60 patients, with intact periosteum in 25 patients, partial periosteum in 13 patients, and resection or absence of the entire periosteum in 12 patients.

Among these 60 patients, 53 (88.33%) demonstrated continuous bone regeneration between mandibular stumps during follow-up. The mean time period between surgery and the first postoperative evidence of spontaneous bone regeneration was 2.33 months (range: 17 days to 6 months). In 39 patients, the mean recovery time for mandibular continuity was 19.87 months, ranging from 3 weeks to 108 months.

Most of the included patients underwent fixation of the mandibular stump after mandibulectomy. Arch bar combined with intermaxillary fixation and reconstruction plate were the most common methods of fixation. Nevertheless, 13 patients without any fixation still demonstrated bone regeneration and eventually achieved mandibular continuity. The mean mandibular continuity recovery time in these 13 patients was 30.29 months, ranging from 3 weeks to 7 years (Table 1).

- Study quality assessment

**Figure 2:**
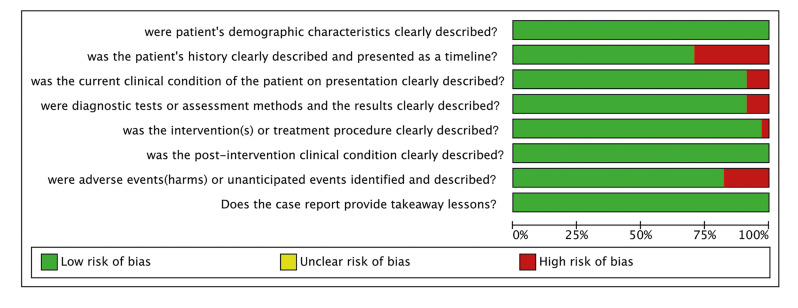
Risk of bias for case reports – graph.

**Figure 3:**
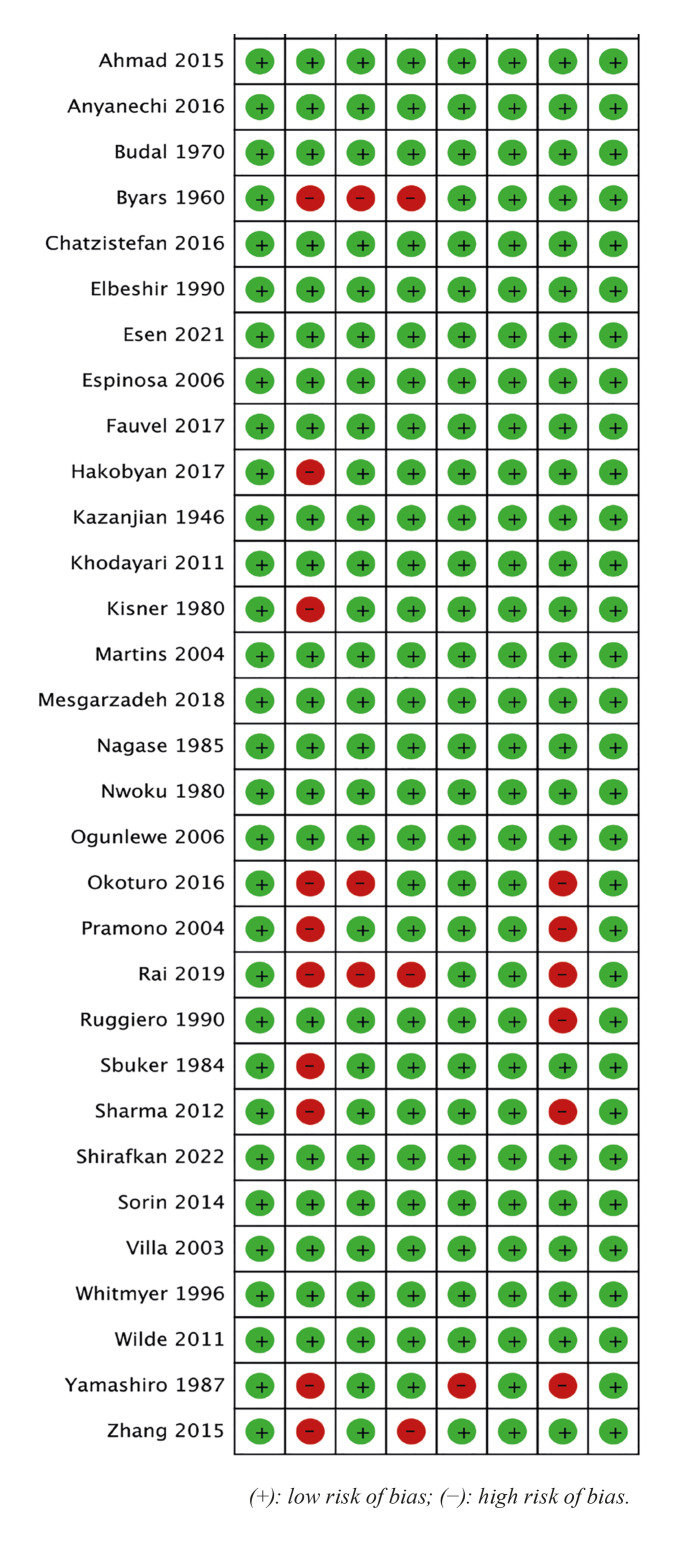
Risk of bias for case reports – summary.

The JBI critical appraisal checklist for case reports was used to appraise the methodological quality of the included articles (Fig. 2, Fig. 3). All 34 articles had a low risk of bias, with a mean score of 93.38% (Table 2). All the included studies clearly described patients’ demographic characteristics, post-intervention clinical condition, and the takeaway lesson (n = 34). Basic patient history was presented in 24 studies, while the current clinical condition, diagnostic tests or assessment methods, and their results were clearly described in 31 studies. Clear descriptions of the intervention or treatment procedure and adverse or unanticipated events were provided in 33 and 28 studies, respectively.

## Discussion

The mean age of the included patients was 20.81 years, with 88.3% (n = 53/60) of the patients being less than 40 years old. It is well-known that spontaneous bone regeneration after mandibulectomy primarily depends on the residual growth potential of the mandible. The mandibular growth peak has been identified as between 14 and 16 years of age in several previous studies (38). The length and dimensional structure of mandible achieve stability at the age of 17-20 years (38-39). This indicates that mandible’s self-healing ability is most active during this period. The fact that the majority of eligible patients in this study were teenagers or young adults also supports this viewpoint. Therefore, mandibulectomy without simultaneous rehabilitation in young patients could potentially lead to spontaneous bone regeneration, with favorable functional and esthetic outcomes.

More than a half of our included patients (56.7%) underwent ostectomy for benign tumors of the mandible. It is well-established that benign neoplasms and low-grade malignancies of the mandible tend to remain confined within the periosteal envelop, without periosteal invasion. In these patients, the adjoining periosteum is often preserved during segmental mandibulectomy. Previous studies have demonstrated that mandibular growth in different regions of the mandible is associated with various processes (38). An important type of mandibular growth presents as continuous apposition and resorption, resulting in bone remodeling at periosteal and endosteal interfaces (39). In other words, if a significant portion of the periosteum is preserved at the surgical site, subsequent spontaneous bone regeneration is possible.

Among the cases in this study that included a description of the periosteum status, periosteum was completely or partially preserved in more than 70% of the cases (25 and 13 patients, respectively). Based on the belief that periosteum is the primary source of osteogenic tissue and that abundance of mesenchymal and osteoprogenitor cells in the periosteum contributes to mandibular regeneration, most authors recommend preserving the uninvaded periosteum during segmental mandibulectomy (16).

Notably, spontaneous bony regeneration occurred even in the absence of periosteum in 12 cases. This may be explained by another type of mandibular growth involving cartilaginous osteogenesis of the condyle and ramus (38). During embryonic development, Meckel’s cartilage, angular cartilage, coronoid cartilage, and condylar cartilage act as principal ossification centers for mandible formation (39). After the growth peak, chondral progenitor cells may remain in all these ossification centers, which may lead to bony regeneration following injury.

In this review, operative approach was specified in 25 patients, including intraoral approach in 12 patients, extraoral approach in 8 patients, and a combined approach in 5 patients. The operative approach did not seem to affect spontaneous bone regeneration after segmental mandibulectomy. However, the choice of operative route may result in undermining of the periosteal or chondral osteogenic potential.

Based on Jewer’s HCL mandibular defect classification (40), 39 cases involved L type defects, 12 cases involved H type defects, and 15 cases involved C type defects. Overall, the study encompassed all anatomical sites of the mandible, indicating that all types of mandibular segmental defects may have spontaneous regeneration potential.

Most of the patients (n = 47) in this study underwent fixation of the mandibular stump after mandibulectomy. Rigid fixation is considered essential for recovery in cases of mandibular fractures. Similarly, immobilization of the mandibular stump after ostectomy is widely acknowledged as an important factor for restoring the occlusion and spontaneous bone regeneration (35). A sTable defect space and physiological environment favor the activation of periosteal and chondral osteogenesis. We found that the mean mandibular continuity recovery time (30.29 months) in the 13 patients without stump fixation was significantly greater than the overall average (19.87 months). This indicates that mandibular stump fixation after mandibulectomy may be necessary for spontaneous bone regeneration.

In this review, the mean duration between surgery and the first postoperative evidence of spontaneous bone regeneration in 41 patients was 2.33 months. Meanwhile, the average recovery time of mandibular continuity was 19.87 months. The present study is the first to describe the gross rate of spontaneous bone regeneration after mandibulectomy. Based on these findings, we recommend that the first radiological examination should be performed 3 months postoperatively to assess spontaneous bone regeneration. Furthermore, regular follow-ups and clinical examinations should be continued for at least 1.5 years after mandibulectomy to determine whether mandibular continuity is achieved.

This systematic review had some limitations that should be considered when interpreting the results. This systematic review included a sample of 60 patients, which was relatively small for drawing robust conclusions. Despite searching various databases without any time or language restrictions, a potential publication bias may still exist. Furthermore, the absence of detailed clinical information in some of the included studies may have influenced final data synthesis.

## Conclusions

This systematic review demonstrated that spontaneous bone regeneration could occur in segmental mandibular defects, particularly in young patients with completely or partially intact periosteum and rigid fixation of the mandibular stump. Nevertheless, further animal experiments and large-scale prospective clinical studies are required to validate our findings.

## Figures and Tables

**Table 1:** Characteristics of the patients included in the systematic review.

Author	Year	Age	Gender	Anatomic location	Etiology	Operative approach	Periosteal status	Stump immobilization	Mandibular continuity recovery	Duration of postoperative spontaneous bone formation	Mandibular continuity recovery time
Kazanjian	1946	15 y	Male	Left mandibular angle and ramus	Ossifyig fibroma	Intraoral	Completely preserved	No	Yes	3 m	3 m
Byars	1960	9 y	Male	Left mandibular body	Fibrous dysplasia	Extraoral	Partially preserved	Yes (Kirschner wire)	Yes	5 w	5 m
		8 y	Female	Right mandible	Ossifyig fibroma	NM	Completely preserved	Yes (Kirschner wire)	Yes	5 w	5 m
Budal	1970	35 y	Female	Mandibular body	Osteofibroma	Intraoral	Partially preserved	No	Yes	2 w	3 w
Adekeye	1977	15 y	Male	Anterior mandible	Ameloblastoma	Extraoral	Completely preserved	No	Yes	NM	7 m
Nwoku	1980	15 y	Male	Left mandible	Ameloblastic fibroma	NM	NM	Yes (IMF)	Yes	NM	6 m
		12 y	Female	Left mandible	Ossifying fibroma	NM	Partially preserved	Yes (IMF)	Yes	NM	6 m
Kisner	1980	12 y	Male	Anterior mandible	Gunshot injury	Combined	Absent	Yes (Kirschner wire)	Yes	NM	10 m
Sbuker	1984	7 y	Male	Left mandible	Avulsion wound	Combined	Absent	Yes (Kirschner wire)	Yes	NM	2.5 y
Nagase	1985	12 y	Male	Left mandible	Ameloblastoma	Extraoral	Partially preserved	Yes (IMF)	Yes	2 w	3 m
Yamashiro	1987	68 y	Male	Mandible	Osteoradionecrosis	Intraoral	NM	No	Yes	NM	7 y
Elbeshir	1990	32 y	Female	Left mandible	Osteomyelitis	NM	Completely preserved	No	Yes	1 m	5 m
Ruggiero	1990	27 y	Male	Right Mandibular ramus	Keratocyst	Extraoral	Partially preserved	No	Yes	NM	9 y
		27 y	Male	Right mandible	Ewing's sarcoma	Extraoral	Partially preserved	Yes (IMF)	Yes	NM	18 m
Whitmyer	1996	9 y	Female	Right Mandibular body	Osteosarcoma	Intraoral	NM	Yes (reconstruction plate)	Yes	3 m	1.5y
Villa	2003	58 y	Female	Mandibular body	Blast injury	Combined	Absent	Yes (extraoral fixation device)	Yes	NM	6 m
Pramono	2004	6 y	Male	Right mandible	Ameloblastoma	NM	Completely preserved	Yes (reconstruction plate)	Yes	NM	6 m
Martins	2004	14 y	Male	Whole Mandibular body	Ossifying fibroma	Extraoral	Partial preserved	No	Yes	NM	2 y
Ogunlewe	2006	13 y	Male	Whole mandible	Ameloblastoma	Intraoral	Completely preserved	Yes (arch bar)	Yes	2.5 m	1 y
Espinosa	2006	7 y	Male	Left Mandibular body	Juvenile ossifying fibroma	Extraoral	Completely preserved	Yes (reconstruction plate)	Yes	NM	6 m
Khodayari	2011	19 y	Male	Left mandible	Odontogenic keratocyst	Intraoral	Completely preserved	Yes (reconstruction plate)	Yes	NM	1y
Wilde	2011	55 y	Female	Right mandible	MRONJ	Extraoral	Completely preserved	Yes (reconstruction plate)	Yes	NM	1y
Abdulai	2012	12 y	Female	Mandibular body	Ameloblastoma	Extraoral	Completely preserved	No	Yes	6 w	6 y
Adebayo	2012	16 y	Male	Left mandible	Giant odontogenic myxoma	Extraoral	NM	Yes (IMF)	Yes	4 w	1 y
Sharma	2012	11 y	Male	Left mandible	Juvenile ossifying fibroma	NM	Completely preserved	Yes (distraction device)	Yes	2 m	18 m
		7 y	Female	Right Mandibular ramus	Aneurysmal bone cyst	NM	Completely preserved	No	No	2 m	12 m
		12 y	Male	Right body of mandible	Hemangioendothelioma	NM	Completely preserved	Yes (reconstruction plate)	Yes	3 m	6 m
		6 y	Male	Right body of mandible	Juvenile ossifying fibroma	NM	Completely preserved	Yes (reconstruction plate)	Yes	3 m	11 m
Sorin	2014	7 y	Male	Right horizontal Mandibular branch	Ewing's sarcoma	NM	NM	Yes (reconstruction plate)	Yes	17 d	15 m
Ahmad	2015	16 y	Male	Left posterior Mandibular body and ramus	Ameloblastoma	Extraoral	Completely preserved	Yes (reconstruction plate)	Yes	4 m	8 m
Zhang	2015	48 y	Female	Left mandible	Ameloblastoma	NM	Partially preserved	No	Yes	9 w	6 m
Anyanechi	2016	16 y	Female	Left mandible	Ameloblastoma	NM	Absent	Yes (IMF)	Yes (10 of 13 patients)	10.5 w	NM
		17 y	Female	Right mandible	Ameloblastoma	NM	Absent	Yes (IMF)		9 w	NM
		19 y	Male	Right mandible	Central giant cell granuloma	NM	Absent	Yes (IMF)		12.4 w	NM
		21 y	Female	Right mandible	Keratocyst	NM	Partially preserved	Yes (IMF)		13 w	NM
		24 y	Male	Left mandible	Ameloblastoma	NM	Absent	Yes (IMF)		13 w	NM
		26 y	Female	Right mandible	Ameloblastoma	NM	Absent	Yes (IMF)		9.8 w	NM
		26 y	Male	Midline and left mandible	Ameloblastoma	NM	Absent	Yes (IMF)		14.3 w	NM
		27 y	Female	Midline mandible	Ameloblastoma	NM	Absent	Yes (IMF)		14.7 w	NM
		30 y	Male	Left mandible	Ossifying fibroma	NM	Absent	Yes (IMF)		15.8 w	NM
Anyanechi	2016	33 y	Female	Left mandible	Odontogenic myxofibroma	NM	Partially preserved	Yes (IMF)	Yes (10 of 13 patients)	16 w	NM
		37 y	Male	Right mandible	Ameloblastoma	NM	Partially preserved	Yes (IMF)		17 w	NM
		39 y	Male	Left mandible	Ossifying fibroma	NM	Partially preserved	Yes (IMF)		16.8 w	NM
		51 y	Male	Right mandible	Ameloblastoma	NM	Partially preserved	Yes (IMF)		17 w	NM
Okoturo	2016	4 y	Male	Mandible	Giant cell granuloma	NM	Completely preserved	Yes (IMF)	Yes (6 of 8 patients)	3 w	NM
		8 y	Female	Mandible	Giant cell granuloma	NM	Completely preserved	Yes (arch bar + IMF)		3 w	NM
		10 y	Male	Mandible	Ameloblastoma	NM	Completely preserved	Yes (arch bar + IMF)		3 w	NM
		11 y	Male	Mandible	Fibromyxoma	NM	Completely preserved	Yes (arch bar + IMF)		5 w	NM
		12 y	Female	Mandible	Ameloblastoma	NM	Completely preserved	Yes (plates + IMF)		3 w	NM
		14 y	Male	Mandible	Ameloblastoma	NM	Completely preserved	Yes (IMF)		3.5 w	NM
		15 y	Male	Mandible	Ameloblastoma	NM	Completely preserved	Yes (arch bar + IMF)		5 w	NM
		12 y	Male	Mandible	Ameloblastoma	NM	Completely preserved	Yes (plates + IMF)		3 w	NM
Chatzistefanou	2016	2 y	Female	Right mandible	Ewing's sarcoma	Extraoral	Partially preserved	No	Yes	NM	2 y
Fauvel	2017	5 y	Male	Mandible	Juvenile ossifying fibroma	Intraoral	Completely preserved	Yes (IMF)	Yes	NM	1 y
Hakobyan	2017	48 y	Male	Mandible	MRONJ	Combined	NM	No	Yes	1 m	3 y
Mesgarzadeh	2018	9 y	Male	Left body of the mandible	Myxofibrosarcoma	Intraoral	NM	Yes (reconstruction plate + IMF)	Yes	8 w	7y
Rai	2019	17 y	Male	Right mandible	Ameloblastic fibroma	NM	NM	Yes (reconstruction plate)	Yes	6 m	3 y
		8 m	Male	Anterior mandible	Melanotic neuroectodermal tumor of infancy	NM	NM	No	Yes	3 m	1 y
Esen	2021	73 y	Female	Right mandible	MRONJ	Intraoral	Completely preserved	Yes (reconstruction plate)	Yes	NM	1 y
Shirafkan	2022	32 y	Female	Left mandible	Cementoossifying fibroma	Intraoral	Completely preserved	Yes (reconstruction plate + IMF)	Yes	NM	1 y

Abbreviations: Year, y; Month, m; Week, w; Day, d; NM, not mentioned; IMF, intermaxillary fixation.

**Table 2:** JBI Critical Appraisal Checklist score of the included articles.

Study name	1	2	3	4	5	6	7	8	Total score %
Abdulai, 2012	Y	Y	Y	Y	Y	Y	Y	Y	100
Adebayo, 2012	Y	Y	Y	Y	Y	Y	Y	Y	100
Adekeye, 1977	Y	Y	Y	Y	Y	Y	Y	Y	100
Ahmad, 2015	Y	Y	Y	Y	Y	Y	Y	Y	100
Anyanechi, 2016	Y	Y	Y	Y	Y	Y	Y	Y	100
Budal, 1970	Y	Y	Y	Y	Y	Y	Y	Y	100
Byars, 1960	Y	N	N	N	Y	Y	Y	Y	62.5
Chatzistefan, 2016	Y	Y	Y	Y	Y	Y	Y	Y	100
Elbeshir, 1990	Y	Y	Y	Y	Y	Y	Y	Y	100
Esen, 2021	Y	Y	Y	Y	Y	Y	Y	Y	100
Espinosa, 2006	Y	Y	Y	Y	Y	Y	Y	Y	100
Fauvel, 2017	Y	Y	Y	Y	Y	Y	Y	Y	100
Hakobyan, 2017	Y	N	Y	Y	Y	Y	Y	Y	87.5
Kazanjian, 1946	Y	Y	Y	Y	Y	Y	Y	Y	100
Khodayari, 2011	Y	Y	Y	Y	Y	Y	Y	Y	100
Kisner, 1980	Y	N	Y	Y	Y	Y	Y	Y	87.5
Martins, 2004	Y	Y	Y	Y	Y	Y	Y	Y	100
Mesgarzadeh, 2018	Y	Y	Y	Y	Y	Y	Y	Y	100
Nagase, 1985	Y	Y	Y	Y	Y	Y	Y	Y	100
Nwoku, 1980	Y	Y	Y	Y	Y	Y	Y	Y	100
Ogunlewe, 2006	Y	Y	Y	Y	Y	Y	Y	Y	100
Okoturo, 2016	Y	N	N	Y	Y	Y	Y	Y	75
Pramono, 2004	Y	N	Y	Y	Y	Y	Y	Y	87.5
Rai, 2019	Y	N	N	N	Y	Y	Y	Y	62.5
Ruggiero, 1990	Y	Y	Y	Y	Y	Y	Y	Y	100
Sbuker, 1984	Y	N	Y	Y	Y	Y	Y	Y	87.5
Sharma, 2012	Y	N	Y	Y	Y	Y	Y	Y	87.5
Shirafkan, 2022	Y	Y	Y	Y	Y	Y	Y	Y	100
Sorin, 2014	Y	Y	Y	Y	Y	Y	Y	Y	100
Villa, 2003	Y	Y	Y	Y	Y	Y	Y	Y	100
Whitmyer, 1996	Y	Y	Y	Y	Y	Y	Y	Y	100
Wilde, 2011	Y	Y	Y	Y	Y	Y	Y	Y	100
Yamashiro, 1987	Y	N	Y	Y	N	Y	N	Y	62.5
Zhang, 2015	Y	N	Y	N	Y	Y	Y	Y	75

## Data Availability

The data that support the findings of this study are available on request from the Correspondence. The data are not publicly available due to privacy or ethical restrictions.
